# Osmosis-Based Pressure Generation: Dynamics and Application

**DOI:** 10.1371/journal.pone.0091350

**Published:** 2014-03-10

**Authors:** Brandon R. Bruhn, Thomas B. H. Schroeder, Suyi Li, Yazan N. Billeh, K. W. Wang, Michael Mayer

**Affiliations:** 1 Department of Biomedical Engineering, University of Michigan, Ann Arbor, Michigan, United States of America; 2 Department of Chemical Engineering, University of Michigan, Ann Arbor, Michigan, United States of America; 3 Department of Mechanical Engineering, University of Michigan, Ann Arbor, Michigan, United States of America; Georgia State University, United States of America

## Abstract

This paper describes osmotically-driven pressure generation in a membrane-bound compartment while taking into account volume expansion, solute dilution, surface area to volume ratio, membrane hydraulic permeability, and changes in osmotic gradient, bulk modulus, and degree of membrane fouling. The emphasis lies on the dynamics of pressure generation; these dynamics have not previously been described in detail. Experimental results are compared to and supported by numerical simulations, which we make accessible as an open source tool. This approach reveals unintuitive results about the quantitative dependence of the speed of pressure generation on the relevant and interdependent parameters that will be encountered in most osmotically-driven pressure generators. For instance, restricting the volume expansion of a compartment allows it to generate its first 5 kPa of pressure seven times faster than without a restraint. In addition, this dynamics study shows that plants are near-ideal osmotic pressure generators, as they are composed of many small compartments with large surface area to volume ratios and strong cell wall reinforcements. Finally, we demonstrate two applications of an osmosis-based pressure generator: actuation of a soft robot and continuous volume delivery over long periods of time. Both applications do not need an external power source but rather take advantage of the energy released upon watering the pressure generators.

## Introduction

**Figure 1:**
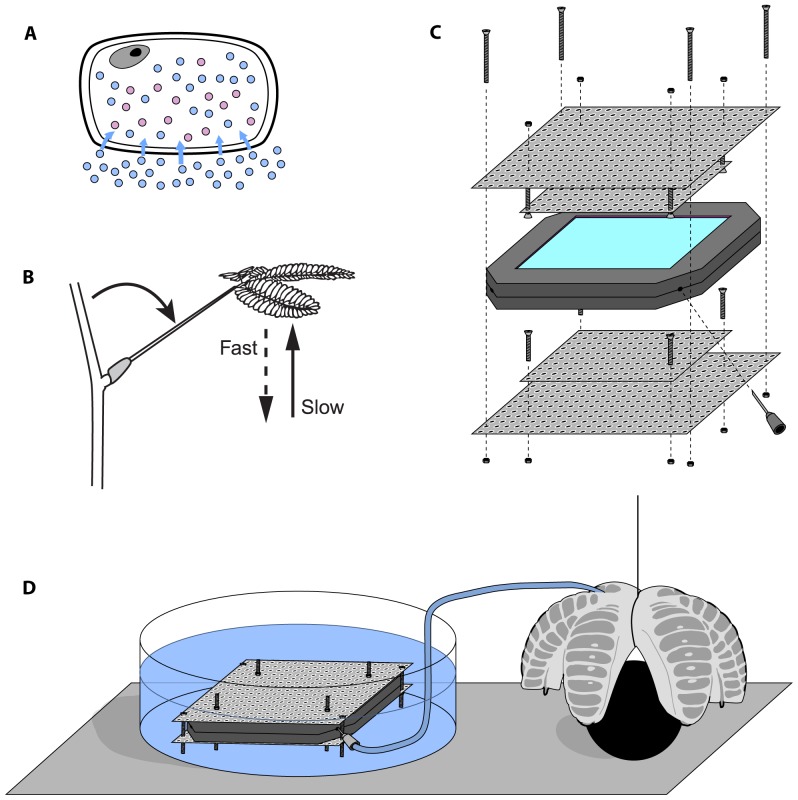
Biological and bioinspired pressure generation and actuation. **A**. Generation of turgor pressure due to an osmotic gradient across the semi-permeable cell membrane of a plant. Blue circles represent water molecules; red circles represent solute molecules. **B**. The nastic motion of *mimosa pudica*. Adapted from Dumais and Forterre, 2012 [52]. **C**. Prototype pressure generator consisting of a restrained dialysis cassette filled with working fluid used in this work. **D**. Schematic of the pressure generator being used to actuate a soft robotic gripper.

The osmotic pressure gradient across a semipermeable membrane separating compartments of differing solute concentrations generates an important driving force in nature. This gradient, represented as *ΔΠ*, quantifies the entropically-driven tendency of the solvent in such systems to flow into the region of higher solute concentration; its value is equal to the pressure gradient that must be applied across the membrane to counteract this flow. As osmotic pressure varies with concentration, a concentration gradient established across a semipermeable membrane will create a pressure gradient if volume expansion on one side of the membrane is limited, as in an enclosed chamber. Plants in particular exploit this phenomenon, extensively employing osmotically-generated pressure gradients (known as turgor) for support and transport of water and solutes (Figure 1A). [1] In addition, certain specialized plants have evolved the ability to change the turgor inside different cells in response to external stimuli. These turgor shifts actuate for instance the opening and closing of guard cells in leaves or the swift nastic motions of the “sensitive plant” *mimosa pudica* in response to touch via clusters of motor cells called pulvini (Figure 1B).

Two classes of osmotic processes are relevant here: forward osmosis (FO) and pressure retarded osmosis (PRO). FO processes are driven by an osmotic gradient *ΔΠ* in the absence of a pressure gradient *ΔP* and are often used to concentrate a solute on one side of a membrane. FO's applications include desalination, purification, and concentration of solute.[2] PRO processes also make use of *ΔΠ* as the dominant driving force; here, *ΔΠ* is used to generate a *ΔP* in the opposite direction. The hydrostatic pressure generated by osmosis can be used to generate electric power using only fresh and salt water; this is a very clean alternative energy source.[3]–[5] A 10 kW power plant has recently been built in Tofte, Norway using PRO technology; a megawatt-scale plant is in the works.[6]

Herein we consider osmosis as a driving force for pressure and shape changes in small devices. Given that approximately 1.3 billion people worldwide lacked access to electricity as of 2012, the development and characterization of devices that can perform tasks without an external power source is critical.[7] Inspired by plants that use osmotic gradients to accomplish mechanical work, we propose that an osmosis-driven device with a high-osmolarity working fluid could similarly serve as a means of liberating usable potential energy from the chemical potential of the fluid via pressure generation simply upon watering the device (Figure 1D). The mathematical model that we used and tested against experimental results revealed a dependency of the speed of pressure generation on volume expansion and membrane area to volume ratio not immediately apparent from the well-known relation between solvent flux and osmotic gradient: [8]–[10]

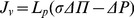
(1)where *J_v_* (m s^−1^) is the volumetric flux of water across the membrane, *L_p_* is the membrane's hydraulic permeability or filtration coefficient (m Pa^−1^ s^−1^), *σ* is the dimensionless osmotic reflection coefficient representing the outward diffusion of the osmotic agent, *ΔΠ* (Pa) is the difference in osmotic pressure between the working fluid compartment and the external solution (e.g. water), and *ΔP* (Pa) is the pressure difference across the membrane.

PRO and FO have largely been explored as industrial processes with parameters (*ΔΠ*, *ΔP*) that are kept constant via circulation mechanisms. By contrast, the pressure generation that occurs in plant cells and our proposed devices is a transient process that builds up a continually increasing pressure gradient in opposition to the osmotically-driven influx of water into the compartment. To our surprise, a review of the literature revealed few previous studies of the *dynamics* of osmotically-driven pressure generation despite the potential usefulness of this information to actuate shape-changing materials and other devices. In 2007, Good *et al*. examined and modeled the displacement of liquid by an expanding (i.e. osmotically swelling) hydrogel compartment in a micropump over time.[11] The modeling, however, was based on the dynamics of polymer swelling rather than osmotic pressure generation. Swelling osmotic compartments have been developed[12]–[14] and modeled[9], [10], [15] for drug delivery applications, but the focus of the modeling has been on the displacement of a pharmaceutical agent rather than internal pressure or volume. Microactuators powered by osmosis have been reported, such as in 2002 by Su *et al*., but a mathematical model of their dynamics has yet to be developed.[16] The work presented here closes this gap.

## Materials and Methods

### General Procedure for Pressure Generation Experiments

**Figure 2:**
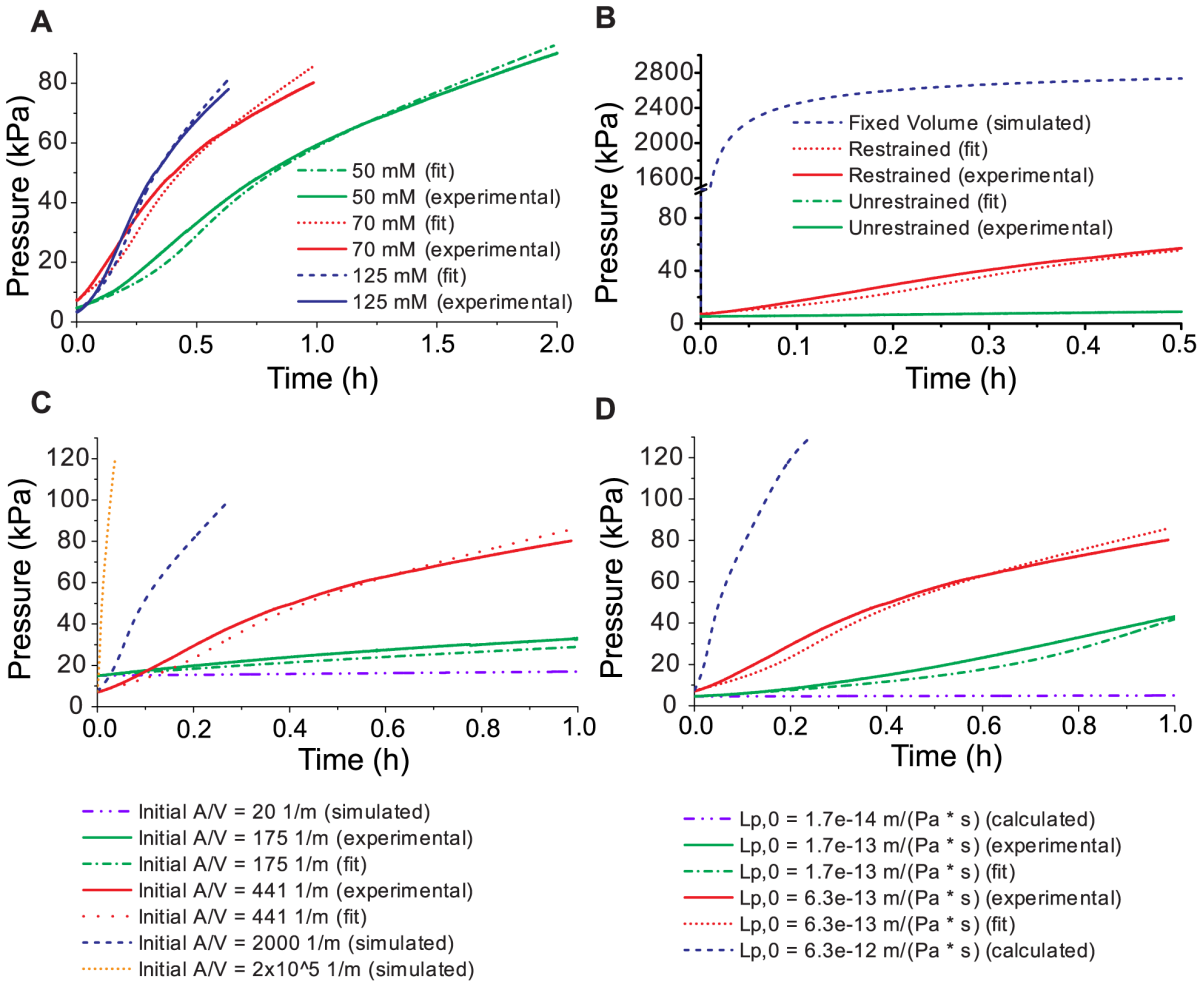
Dynamics of osmotically-driven pressure generation and comparison of the model based on Equation 4 with experimental data. A. Time-dependent pressure generation as a function of PEG-4000 concentration inside the dialysis cassette. B. Time-dependent pressure generation as a function of various mechanical constraints of the volume inside the dialysis cassette. C. Pressure generation dynamics as a function of various initial surface area to volume ratios. The dashed orange curve represents an initial *A/V* ratio taken from extensor cells of the plant *Phaseolus coccineus*.[24] D. Pressure generation dynamics as a function of various *L_p,0_* values.

To provide simple and accessible systems for studying the dynamics of osmotically-driven pressure generation, we constructed a restraint around commercially available 3 mL Slide-A-Lyzer dialysis cassettes (Thermo Scientific) with a molecular weight cutoff (MWCO) of 2,000 Da (Product #66203) or 3,500 Da (Product #66330) using perforated stainless steel plates (see Figure 1C). After bolting the dialysis cassette into the restraint, we hydrated it in water (Millipore Milli-Q purification system) for at least 2 min. We produced a working fluid of the desired concentration by diluting a 50% w/v (125 mM) aqueous poly(ethylene glycol) solution (PEG-4000, Hampton Research, HR2-529) with Milli-Q water, then added 3 mL to the restrained cassette. We attached a pressure transducer (ICU Medical, Transpac IV Disposable Pressure Transducer) to a length of rigid tubing with a needle at the end, filled this transducer setup with working fluid, and inserted the needle into the cassette through one of its ports, taking care to ensure that no air bubbles were allowed into the system. We monitored the pressure continuously via a custom-built LabView VI after connecting the transducer to a DAQ board (National Instruments BNC-2110) and a DC power supply (Hewlett Packard E3612A) at 6 V. Prior to submerging the cassette, we added working fluid via syringe until the internal pressure reached approximately 5 kPa. At this point, we lowered the restrained cassette into a large reservoir containing Milli-Q water to start pressure generation. Recording proceeded until failure of the cassette or of a fluidic connection. For the experiments in Figure 2, we generally used 3 mL cassettes with a 3.5 kDa MWCO and initial PEG concentration of 70 mM. In Figure 2A, we varied the initial PEG concentration; in Figure 2B, we submerged one cassette without restraint; in Figure 2C, we varied the cassette size; in Figure 2D, we varied the MWCO of the dialysis membrane. Note that the initial membrane area to volume ratios for the 3 mL cassettes used were usually approximately 400 m^−1^ while the ratio for 30 mL cassette was approximately 170 m^−1^.

### Pumping Experiment for L_p,0_ Determination

For each MWCO, we restrained and hydrated a 3 mL cassette as described before, filled it slightly over capacity with pure water, and resubmerged it in a water bath. We attached a length of thin, rigid tubing of known diameter to the cassette. The other end of the tubing was connected to a constant-pressure pump (Nanion Suction Control Pro). Upon attachment to the cassette, the excess water from the cassette filled the tubing to a large extent before the meniscus stopped moving. The tubing was laid against a ruler and the pump was set to 30 kPa. The meniscus began moving toward the cassette; when it reached the ruler, its time and position were periodically recorded to obtain a volumetric flux.

### Volume Delivery Experiment

After filling the dialysis cassette slightly over capacity with working fluid (PEG concentration 125 mM), we attached a length of rigid tubing of known volume filled with the same fluid to the cassette via a needle. We suspended the other end of the tubing above a graduated cylinder. We allowed fluid to exit the tube until the pressure inside the overfilled cassette fully equilibrated to atmospheric pressure. We emptied and replaced the graduated cylinder, then wrapped the cylinder opening and tubing loosely with parafilm to slow evaporation. We submerged the cassette in Milli-Q water and periodically recorded the time and volume of fluid in the cylinder.

### Soft Robot Actuation Experiment

We submerged a soft robotic gripper[17] obtained from the Whitesides group in water (Millipore Milli-Q purification system) and put it under vacuum until all air bubbles had escaped. We then fitted the gripper with rigid tubing and a needle filled with Milli-Q water. We prepared the osmotic pressure generator and pressure transducer as described in the pressure generation section, although for this experiment we used a 30 mL Slide-A-Lyzer dialysis cassette (Thermo Scientific) with a molecular weight cutoff of 3,500 Da (Product #66130), pre-filling it with 18 mL of solution. Just prior to initiating pressure generation, we attached the gripper to the cassette. We set up a camera to take images of the gripper every 20 seconds, then lowered the restrained cassette into the water and promptly initiated pressure recording and photography. Recording proceeded until failure of a fluidic connection to the gripper. The gripper depressurized upon failure or disconnection from the cassette.

## Results and Discussion

We constructed prototypical pressure generators using commercially available dialysis cassettes with MWCOs of 2,000 and 3,500 Da and various concentrations of aqueous PEG-4000 as a working fluid. Dialysis cassettes are commercially available and easy to set up as pressure generators; PEG solution was chosen as a working fluid due to its compatibility with the cassettes. We restrained the dialysis membranes of these cassettes with a porous steel screen to delay membrane failure and restrict volume expansion (Figure 1C). Upon submersion, the device generated pressure as shown in Table 1 and Figure 2.

**Table 1:** Dynamics of pressure generation as a function of various parameters. Each row represents one experiment.

Membrane Cutoff (kDa)	Initial volume (10^−6^ m^3^)	Initial *A/V* ratio (m^−1^)^a^	Initial [PEG] (mM)	Initial osmotic pressure (10^6^ Pa)	Time to gain 10 kPa (min)	Time to gain 50 kPa (min)	Calculated *A* (cm^2^)
2.0	5.03	435.4	30	0.38	28	206	21.9
2.0	4.93	444.2	50	1.31	26	132	23.0
2.0	4.73	463.0	70	3.09	23	75	32.3
2.0	4.75	461.1	70	3.09	14	58	41.3
2.0	4.52	484.5	125	14.73	26	53	41.4
2.0	4.66	470.0	125	14.73	10	38	51.9
2.0 [unrestrained]	6.47	338.5	70	3.09	134	[n/a] b	18.9
3.5	5.98	366.2	30	0.38	15	104	21.2
3.5	5.92	369.9	50	1.31	14	54	20.4
3.5	5.99	365.6	70	3.09	6	30	26.4
3.5	6.28	348.7	70	3.09	5	[n/a] b	21.4
3.5	5.88	372.4	125	14.73	6	22	22.3
3.5	5.86	369.9	125	14.73	6	[n/a] b	23.9
3.5 [30 mL]	35.2	170.5	70	3.09	27	311	61.5
3.5 [unrestrained]	6.97	314.2	70	3.09	[n/a] b	[n/a] b	36.2
Pulvinus extensor cell	3.23×10^−7^ c	6.27×10^4^ c	c	1.98^d^	5^d^	8^d^	2.03×10^−4^ c

a Initial membrane area was assumed to be 21.9 cm^2^ for 3 mL cassettes, 60.0 cm^2^ for 30 mL.

b [n/a] indicates failure before pressure point.

c Extensor cell surface area and volume taken from *Phaseolus coccineus* [24]; *L_p_* (3.63×10^−14^ m Pa^−1^ s^−1^) taken from *Samanea Saman* in the evening.[25]

d Osmolality of an active pulvinar cell ≈1000 mOsm/kg.[26] This calculation was made using the van't Hoff equation rather than Equation S1 in Appendix S1 as the osmotic agents in a plant cell are primarily sugars and ions rather than polymers. The fouling factor was assumed to be zero for the same reason.

To characterize the dynamics of the system, we modeled the pressure generation by using two differential equations for the change in mass within the cassette over time. First,
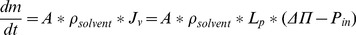
(2)where *A* (m^2^) is the total membrane area and *ρ_solvent_* (kg m^−3^) is the density of water. This equation assumes that the reflection coefficient *σ* equals one, representing a leak-free membrane over the course of the entire experiment. For ideal solutions, *Π* is known to vary linearly with concentration according to the van't Hoff relation. However, polymer solutions often deviate from this linearity; the slope of *Π* versus concentration increases as a function of concentration.[18] Using a freezing-point depression osmometer, we established that the osmotic pressure of aqueous PEG-4000 solutions increases as a function of PEG concentration following a third-order polynomial function (*N* = 14, *R* ^2^>0.99, see Appendix S1 and Figure S1). PEG's favorably non-linear osmotic pressure curve and large size make it an attractive osmotic working fluid. A 0.125 M solution of PEG-4000 generates an osmotic driving force comparable to that of a solution of NaCl at its solubility limit of 5.28 M [19] while being easily retained by a dialysis cassette with a MWCO in the kDa range.

Additionally, we determined from a series of volume delivery experiments that the effective *L_p_* decreases drastically with increasing PEG concentration (see Appendix S2 and Figure S2). We conjecture that PEG molecules bind to the pores in the dialysis membrane, impeding the flux of water. Such membrane fouling is one of two phenomena observed in industrial osmotic processes that could cause a similar reduction in *L_p_* – the other, concentration polarization, is considered negligible across dialysis membranes such as ours (see Appendix S3).[20], [21] We therefore split *L_p_* into two parameters according to *L_p_* = *L_p,0_* (1 - *f*), where *L_p,0_* is the *L_p_* value in the absence of PEG and *f* is a “fouling factor” that represents the percentage of blocked pores. We found that *f* varies as a function of PEG concentration and is described well by the Hill equation for cooperative binding (R^2^>0.98, see Appendix S3).

Second, from the definition of bulk modulus *K* (Pa), while *K*>>*P_in_* we obtain
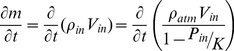
(3)where “in” denotes a characteristic of the cassette's interior, *ρ_atm_* (kg m^−3^) is the density of the working fluid at atmospheric pressure. We determined the bulk modulus of aqueous PEG solutions with different concentrations and at various pressures by measuring the velocity of a pressure wave in solution and found that *K* follows a second-order polynomial function with respect to PEG concentration and varies linearly with pressure (*N* = 58, *R* ^2^ = 0.93, see Appendix S4 and Figures S3 and S4). Finally, we measured the pressure-dependent volume expansion *V_in_*(*P_in_*) for each type of cassette and restraint using a procedure described in Appendix S5. Considering volume expansion as a function of pressure ensures that time-dependent changes in the PEG concentration (i.e. solute dilution) as well as the resulting implications for *ΔΠ*, *K*, *f*, and *P_in_* are accounted for.

Combining Equation 2 and Equation 3 yields a governing equation:
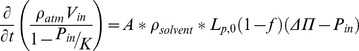
(4)


This differential equation has no simple analytical solution for *P_in_* versus *t* even when all parameters are fixed, so we solved Equation 4 numerically using a variable step Runge-Kutta method in MATLAB (the ode45 function). In order to solve for *P_in_*(*t*), we needed to find values or functional relations for each of the other parameters in Equation 4. We used the *V_in_*(*P_in_*) relationship and the initial PEG concentration to obtain an expression for the concentration of the working fluid, which is simply the ratio of the number of moles of PEG inside the cassette (assumed to be constant) over the volume of the cassette, *V_in_*(*P_in_*). Since the *K, f*, and *ΔΠ* functions determined previously are dependent on concentration, it was then possible to express each as a function of *P_in_*. We used the density of water [22] for *ρ_solvent_* and a density calculated as a function of initial PEG concentration at atmospheric pressure for *ρ_atm_* [23] We assumed that the membrane hydraulic permeability *L_p,0_*, which we obtained in a manner described in Appendix S3, would stay constant over the course of the experiment. The *L_p,0_* value obtained for dialysis cassettes with a MWCO of 3.5 kDa was 6.26×10^−13^ m Pa^−1^ s^−1^, over three times the value obtained for cassettes with a MWCO of 2.0 kDa, which had an *L_p,0_* of 1.70×10^−13^ m Pa^−1^ s^−1^. This analysis confirms the expectation that membranes with a large MWCO are more permeable than membranes with a smaller MWCO.

After defining these values and relations, the parameter most difficult to obtain a definite value for is *A*, as it is unclear from observation whether the available membrane area increases with bulging upon pressurization or decreases due to blocking by the restraint. To determine *A* for each experiment, we ran a fitting script with a set of experimental data in which the area was allowed to vary. The script returns the *A* value that minimizes the residual sum of squares between the experimental data of *P_in_* as a function of time and the pressure generation curve predicted by Equation 4. In this way, we are able to determine *A* based on *P_in_*(*t*) data or predict a *P_in_*(*t*) curve for a given *A* in compartments of known *L_p,0_*, fouling behavior, and *V_in_*(*P_in_*) relationship. For 3 mL cassettes, the mean area of the 2.0 kDa MWCO membranes was determined to be 35.3±11.7 cm^2^ while the mean area of the 3.5 kDa MWCO membranes is 22.1±2.5 cm^2^. For reference, the estimated bulging membrane area is approximately 21.9 cm^2^, so the calculated *A* values are in reasonably good agreement, especially for the 3.5 kDa MWCO cassettes. These values indicate that the steel mesh does not reduce the membrane area available for flux.

The variation between the *A* values shown in Table 1 that were obtained by fitting data from experiments using membranes of the same type is likely a result of simplifications in our model's assumptions. Since *A* is the only fitting parameter, any discrepancy between our modeled *P_in_*(*t*) curve and the experimental data will be reflected in the best-fit value of *A*. Hence, *A* may vary between experiments as it compensates for a number of different factors. Commercial dialysis cassettes are not designed to be pressurized; we had to assemble and disassemble an external reinforcement for experiments. As a result, *V_in_*(*P_in_*) may vary between cassettes of the same type, but the model does not account for such variation. Further, the possible expansion of dialysis membranes upon pressurization as a function of time could increase the effective MWCO of the membrane. Our model assumes that *L_p,0_* and *A* remain constant over the course of the experiment and that the reflection coefficient *σ* remains 1. The polynomial equation for *ΔΠ* as a function of PEG concentration may also introduce some error at concentrations above 80 mM, which are above the range of our osmometer. At low concentrations, the *ΔΠ* driving force is less dominant than in experiments using a concentrated PEG solution, so artifacts caused by small variations in the experimental setup such as air bubbles or slight inaccuracies in initial volume and concentration would have a more noticeable effect. Considering that one or several of these factors may influence the determination of *A*, the observed variations of *A* seem to be within a reasonable range and are generally accurate within a factor of two.

Solving Equation 4 for *P_in_*(*t*) yielded excellent fits of pressure generation curves to the experimental data (Figure 2A). The agreement between the data and the fits validates the approach and shows that solving Equation 4 iteratively makes it possible to predict the pressure generation dynamics for a range of osmotic driving forces and relevant parameters. For example, we were able to predict the dynamics of osmotic pressure generation in chambers with the dimensions and *L_p_* values of plant cells and compare them to our systems; see Figure 2C and Figure 2D. This comparison revealed, for instance, the importance of restraining the cassettes as shown in Figure 2B: restricting *V_in_*(*P_in_*) accelerates pressure generation dramatically. The hypothetical constant volume case would be capable of generating megapascals of pressure within seconds. For practical reasons, a constant *V_in_*(*P_in_*) relationship is more readily attainable on a small scale. In addition, scaling down the compartment for the working fluid increases the initial membrane-area-to-volume ratio (*A*/*V*) of the system, as *A*/*V* for any object is inversely proportional to characteristic length. A high *A*/*V* value is beneficial for rapid pressure generation as displayed in Figure 2C: the projected pressure generation curve using the *A*/*V* value of plant motor cells (extensors in *Phaseolus coccineus*)[24] increases so quickly that the curve appears completely vertical when plotted on the timescale shown in the figure.

**Figure 3:**
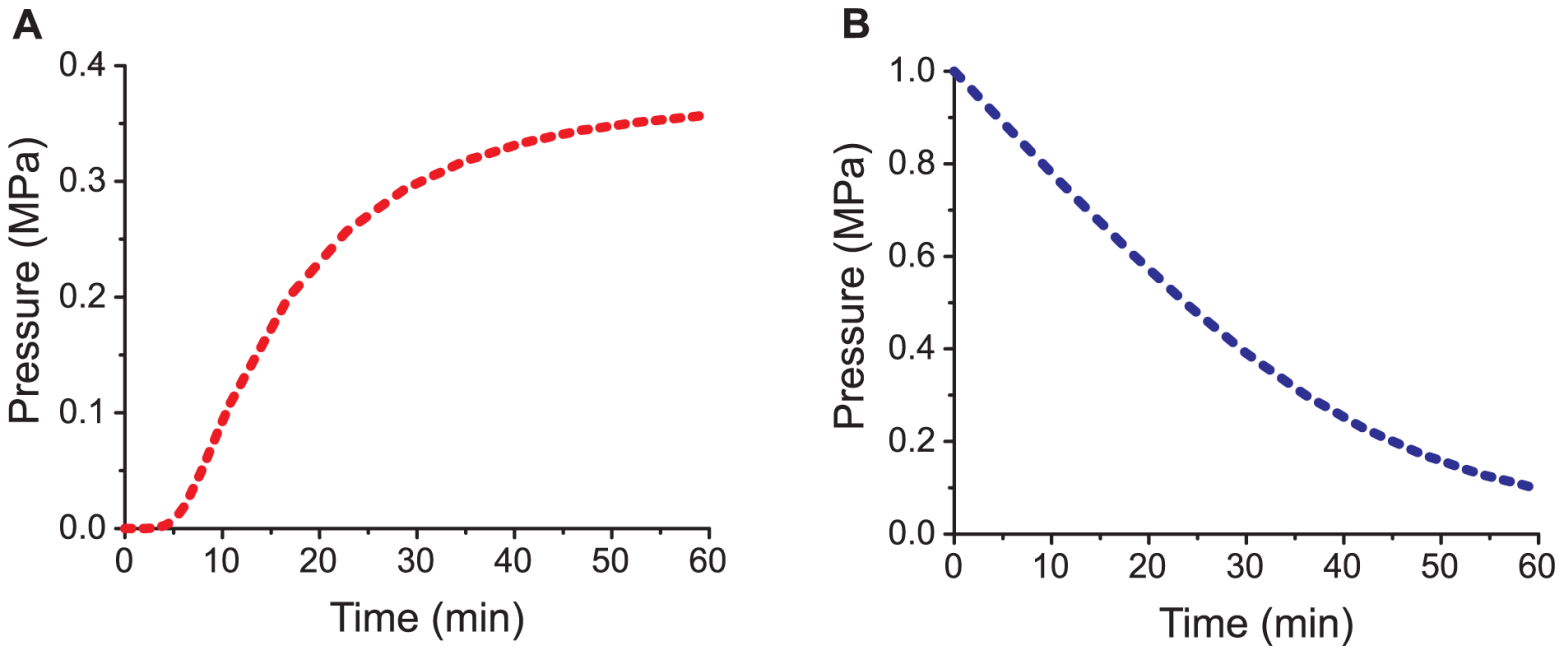
Dynamics of pressure buildup and release in plant extensor cells as calculated withEquation 4 and parameters based on literature values. The *L_p,0_* value used in the calculations was 3.6×10^−14^ m Pa^−1^ s^−1^ [25], the cell volume was 3.23×10^−13^ m^3^, the surface area was 2.02×10^−8^ (both estimated from [24]), and *V_in_(P_in_)* was *V_in_ = *(3.23×10^−13^)exp[1.06×10^−6^ *P_in_*+0.047 ln(*P_in_*)] (developed from literature bulk elastic modulus values[24] and the definition of bulk elastic modulus[27]). In these calculations *ΔΠ* followed the van't Hoff relation and *f* was assumed to be zero. A. Pressure buildup upon sudden introduction of an osmotic gradient with an initial *ΔΠ* of 1 MPa [23] B. Pressure release upon sudden disappearance of an osmotic gradient. Initial *P_in_* was 1 MPa. [23], [24] The difference between the maximum pressure from the pressurization curve and the starting pressure from the depressurization curve can be accounted for by the fact that our model assumes no significant biochemical regulation of osmotic pressure during this fast pressurization. As a consequence, the influx of water leads to a decrease in *ΔΠ*.

Using the dimensions,[24] *L_p_* value,[25] the final *ΔΠ* value[26] from plant pulvini, and a *f* value of zero, along with a *V_in_*(*P_in_*) curve developed from values of bulk elastic modulus in *Phaseolus coccineus* extensor cells [24] and the definition of bulk elastic modulus[27], we used Equation 4 to predict the dynamics of pressure generation in these motor cells (Figure 3A). As summarized in Table 1, this approach suggests that these plant motor cells are able to generate 50 kPa within 8 minutes. This rate is threefold faster than the fastest pressure generators tested here. We point out, however, that the mechanism of motor cell function in mimosa pudica and other plants that undergo fast motions is slightly different than the devices presented here. In fast-moving plants, turgid cells are prefilled with osmotic agents (often potassium and chloride ions) via protein-mediated active transport, an energy-intensive process that takes place on the time scale of minutes to hours.[28] Upon receiving a stimulus, ion channels open in these cells, causing the osmotic agents to equilibrate with the extracellular fluid.[29] This flux eliminates the osmotic driving force necessary to maintain turgor, so the cells depressurize. A demonstration of this effect assuming instantaneous working fluid equilibration is shown in Figure 3B. The same parameters were used as in the calculated *P_in_*(*t*) curve in Figure 3A, but the initial pressure *P_in_*(0) was set to 1 MPa[30], [31] and upon stimulation *ΔΠ* was set to zero. This approach predicts that cells with these characteristics are able to lose 10% of their osmotic pressure (∼100 kPa) in 4.5 minutes. By comparison, *mimosa pudica* extensor cells lose 10% of their pressure within 5 minutes.[32] In principle, strategies for generating an osmotic gradient on-demand such as electro-osmosis and ion transport via membrane proteins could be explored in membrane-bound synthetic actuators and described by our model. The implementation of these methods is beyond the scope of our research.

**Figure 4:**
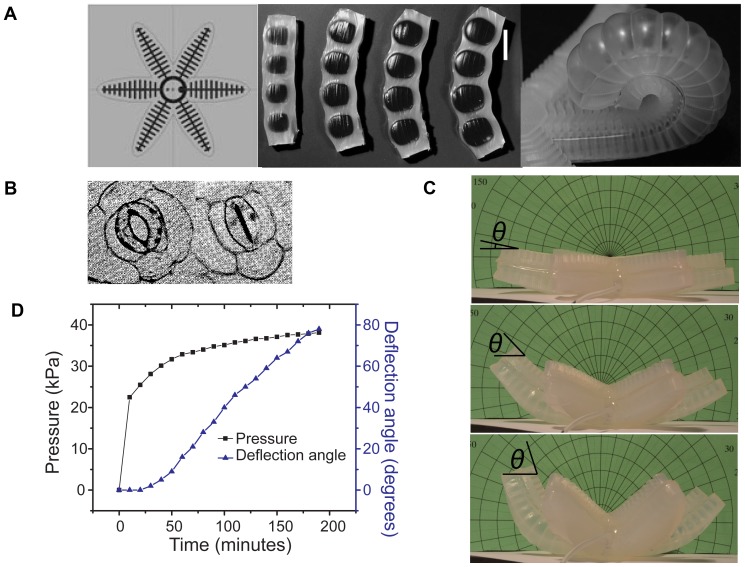
Bioinspired application of an osmotic pressure generator for actuation of a soft robot. **A**. Design concept of a previously described elastomeric soft robot whose arms curve and can grip objects in response to pressurization of parallel channels with boundaries of differing thickness. Adapted from [17]. **B**. Guard cells in their open and closed states. Thick interior walls make guard cells curl when osmotically pressurized, opening stomata. Adapted from [28]. **C**. Actuation of the soft robotic gripper used in this work in response to watering an attached osmotic pressure generator. The extent of deflection is shown after 1, 2, and 3 hours. **D**. System pressure and deflection angle (as defined in C) of the gripper as a function of time.

After modeling the dynamics of pressure generation, we created a bio-inspired actuator system by attaching a 30 mL cassette filled with saturated (125 mM PEG-4000) working fluid to a soft robotic gripper (Figure 1D).[17] Each arm of the gripper is filled with channels that run perpendicular to its length (Figure 4B). The inside walls of the channels are thicker than the outside walls so that when positive pressure is applied, the outer walls deform more than the walls on the interior. When such channels are lined up in a row, the system curves upon pressurization, causing the arms of the gripper to bend inward.[17], [33]–[35] Guard cells open and close plant stomata using a similar mechanism. On each pair of guard cells, the cell wall that faces the pore is thicker than the wall on the outside of the stoma. When coupled with microfibrils that restrict the guard cell circumference to remain relatively constant, this wall architecture causes the cells to curve into an “O” shape when pressure is delivered via osmosis, thus opening the pore in response to external stimuli (Figure 4A).[36]

**Figure 5:**
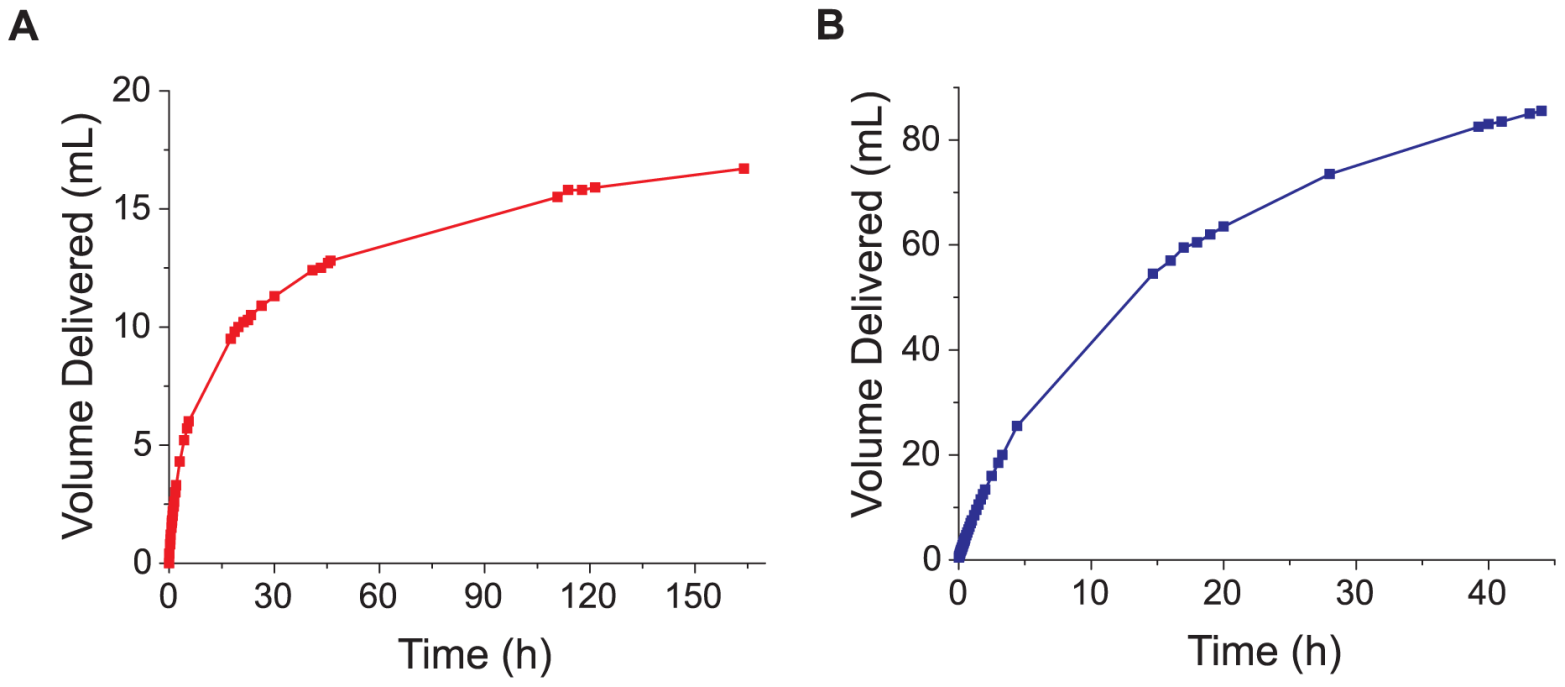
Time-dependent volume delivery using an osmotically-driven pressure generator as a pump. **A**. Cumulative volume delivered by a 3 mL dialysis cassette. **B**. Cumulative volume delivered by a 30 mL cassette. Both cassettes were restrained and filled with a 125 mM PEG-4000 solution and had a MWCO of 3.5 kDa.

We recorded the pressure and deflection angle of the soft robot over time (Figure 5C, Figure 4D, Video S1). Full deflection took approximately three hours, after which the fluidic connection to the gripper sprang a leak. The slow timescale of this actuation was noteworthy, especially considering the quick action of the mimosa plant. Our model confirmed that using larger compartments with lower *A/V* ratios such the prototypes and robot used here slows down pressure generation more dramatically than intuition may suggest (Figure 2C).

Using these relatively large commercially available dialysis cassettes does, however, also have its advantages. First, this system is sufficiently simple and inexpensive that any laboratory could set up a pressure generator with ease – large-scale industrial production of the cassettes should also result in relatively constant values of parameters such as initial *A/V* ratio, *L_p,0_* value, *V_in_*(*P_in_*), etc. Second, near-instantaneous pressure generation is only useful in some scenarios and may not be the most important parameter for a given application. Slow but continuous volume delivery is, for instance, another interesting application of these devices. A 30 mL cassette with a lower initial *A/V* ratio than the 3 mL cassettes typically used in this work took significantly longer to generate pressure as expected (Table 1), but in our volume delivery experiment these larger cassettes were able to pump 86 mL of fluid over approximately two days compared to the smaller cassette which pumped 13 mL in the same time (Figure 5). This kind of extended self-powered pump activity might be particularly attractive for microfluidic applications[37]–[42], which often require flow rates of only nanoliters to microliters per hour. For instance, almost a week after starting an experiment, a generator with 3 mL of initial working fluid was still delivering a flow of greater than 1 µL/min. Additionally, after an initial leveling-off period of approximately 30 h, the flow rate stayed remarkably constant for 3 days, with an average decrease of only 0.4% per hour (Figure 5A).

Restricting volume expansion is another important aspect that should be considered depending on the application of osmotically-driven pressure generators. Allowing the volume of a container to expand causes its internal pressure to rise much more slowly compared to the restricted case (Figure 2B), but a system that swells to accommodate a large volume of pressurized fluid stores more energy than a system with a smaller volume at the same pressure. Consider the pressure-volume work done on the cassette over the course of pressure generation as representative of the energy stored. When we allowed the cassette to expand without a restraint, the first 5 kPa generated from osmosis took five to ten times longer to generate than it did with the restraint. Once this pressure was reached, however, the volume of pressurized fluid in the vessel was much bigger. In this way an unrestrained cassette at 5 kPa stores approximately an order of magnitude more energy than a restrained one at the same pressure. Volume expansion additionally may be desirable if a constant pressure is needed in an osmotic system. During the soft robot experiment, the period from 50 minutes through the end of the experiment saw a relatively constant pressure accompanied by the most rapid deflection of the gripper (Figure 4D). Based on these phenomena, we demonstrate that a volume-expanding element can be used analogously to a capacitor in an electrical system: it can both store energy and stabilize the difference in potential (pressure) between two points. Flexible membranes with fluidic capacitance have been used in microfluidic devices to these ends.[43]–[50]

## Conclusions

The diversity of tasks for which plants use osmosis represents a rich field of potential technological advancements that harness the same driving force to actuate materials and devices.[51] We have shown that an osomotically-driven pressure generator can be constructed from materials that are low-cost and readily available. In addition, the model based on Equation 4 can be used to predict the dynamics of pressure generation for anyone seeking to create devices with the capability to generate pressure or deliver volume for a range of applications. These predictions of the dynamics of such systems cannot readily be achieved without the approach presented here due to the complicated interdependence of time-dependent osmotic pressure generation on initial volume, volume expansion, solute dilution, membrane fouling, initial *A/V* ratio, *K*, and *L_p_*. The MATLAB code used for the calculated pressure generation curves in this paper is available on SourceForge (https://sourceforge.net/projects/osmoticpressurizationdynamics/files/) or in the Supporting Information (Files S1, S2, S3). If an aqueous PEG-4000 solution is used as the working fluid, there are three parameters needed to calculate *P_in_*(*t*): the membrane hydraulic permeability, which is attainable from the pumping experiment described in Appendix S3, the dependence of *f* on PEG concentration, which is attainable from the volume delivery experiments described in Appendix S2, and the dependence of *V_in_*(*P_in_*), which is attainable by measuring the pressure of the device's reservoir while adding known volumes as described in Appendix S5. If the type of cassettes described here are used, these values and relations are described in this paper and do not need to be measured once again. Other solutes can be used as well if the relationship between *ΔΠ* and concentration is known. If precisely determined, *A* can also be input; otherwise the code can load an experimental data set of pressure versus time and find the best-fitting *A* value. We hope other researchers will find this model and the effects shown in Figure 2 useful for defining the dimensions and parameters of pressure generators of their own construction.

One compelling property of the osmotic pressure generators described in this work is that they can generate pressure and fluid flow without access to external power. Simply “watering” the device initiates the process in a manner similar to watering a plant. The novelty of the work presented here – in addition to providing insight into the fundamental and unintuitive time dependence of establishing or releasing turgor pressure in plants (Figures 2 and 3) – lies in a detailed characterization of the dynamics of these engineered osmotic pressure generators such that they may be used in microfluidics and microactuation with predictable outcomes.

## Supporting Information

Figure S1 **Osmotic pressure as a function of PEG concentration.**
(TIF)

Figure S2
**Effects of Membrane Fouling at Increasing PEG Concentrations.** **A**. Effective *L_p_* values calculated from volume delivery experiments as described in Appendix S2 at different PEG concentrations and MWCOs. The *L_p_* value in the absence of PEG, *L_p,0_*, was determined by the procedure in Appendix 3. **B**. The fouling factor *f* at different PEG concentrations and MWCOs. *f* is determined by 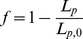 and has been fit with the Hill equation.(TIF)

Figure S3
**Experimental setup for measuring wave velocity.** The Tygon tubing and stainless steel pipe are filled with aqueous PEG solution. An air compressor attached to the accumulator applies a static pressure (*P_in_*) that is measured by a DC pressure transducer (*P_DC_*; 840065, Sper Scientific). Both valves are closed prior to measuring wave velocity to prevent fluid flow. A shaker actuates the piston to create a pressure or sound wave. The time delay between two AC pressure transducers (*P_1_* and *P_2_*) is used to determine wave velocity (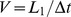). The downstream pressure transducer (*P_2_*) was mounted at a length *L_2_* away from the end of the pipe to ensure that reflections from the pipe/tubing interface did not interfere with the time delay measurements. *L_1_* is 60.7 cm, *L_2_* is 77.0 cm, the inner diameter of the stainless steel pipe is 1.39 cm, and the pipe wall thickness is 3.7 mm (1/2 NPS SCH80).(TIF)

Figure S4 **Bulk modulus calibration and measurements A.** Calibration of bulk modulus measurement apparatus with air. **B**. Bulk modulus measurements of aqueous PEG solutions from the apparatus. The fit lines represent a fit of the entire data set, not just the points from that concentration.
(TIF)

File S1 **MATLAB script for calculation of pressure as a function of time.** This script calculates pressure as a function of time for a pressure generator of specified dimensions and permeability containing an aqueous PEG solution as an osmotic driver. It can also be used to determine the effective membrane area available for flux from a set of experimental data. This code is also available on SourceForge: https://sourceforge.net/projects/osmoticpressurizationdynamics/files/.
(M) Click here for additional data file.

File S2 **Sample data set to accompany File S1.** Contains pressure and time data. This code is also available on SourceForge: https://sourceforge.net/projects/osmoticpressurizationdynamics/files/.
(M) Click here for additional data file.

File S3 **Sample calculation from File S1.** Contains input parameters and resulting calculated pressure and time values. This file is also available on SourceForge: https://sourceforge.net/projects/osmoticpressurizationdynamics/files/.
(XLSX)

Video S1 **Actuation of soft robot attached to a pressure generator.**
(MOV)

Appendix S1 **Osmotic Pressure Measurements.**
(DOCX)

Appendix S2 **Determining** ***L_p_*** **from Volume Delivery.**
(DOCX)

Appendix S3 **Discussion of Correction to** ***L_p_*** **.**
(DOCX)

Appendix S4 **Bulk Modulus Measurements.**
(DOCX)

Appendix S5 **Determination of** ***V_in_*** **(** ***P_in_*** **).**
(DOCX)
