# Supplementary figures and images for: Osmosis-Based Pressure Generation: Dynamics and Application
Source: PLoS One. 2014 Mar 10;9(3):e91350. doi: 10.1371/journal.pone.0091350 (PMC3948862; doi:10.1371/journal.pone.0091350)

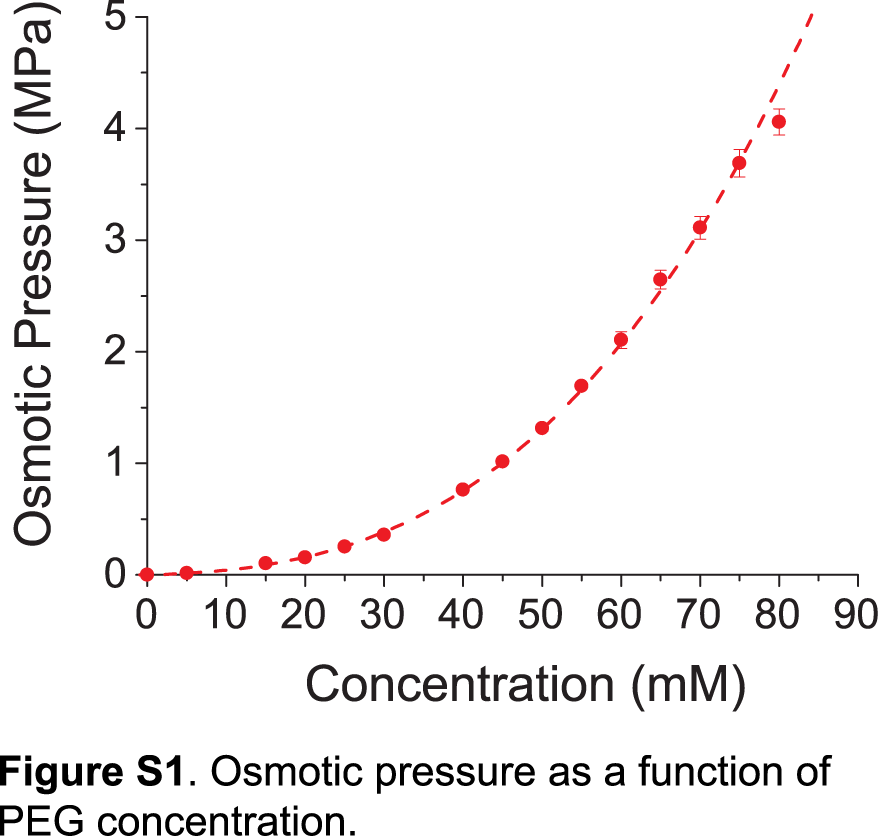

Supplement: Figure S1 — Osmotic pressure as a function of PEG concentration. (TIF) [file pone.0091350.s001.tif]

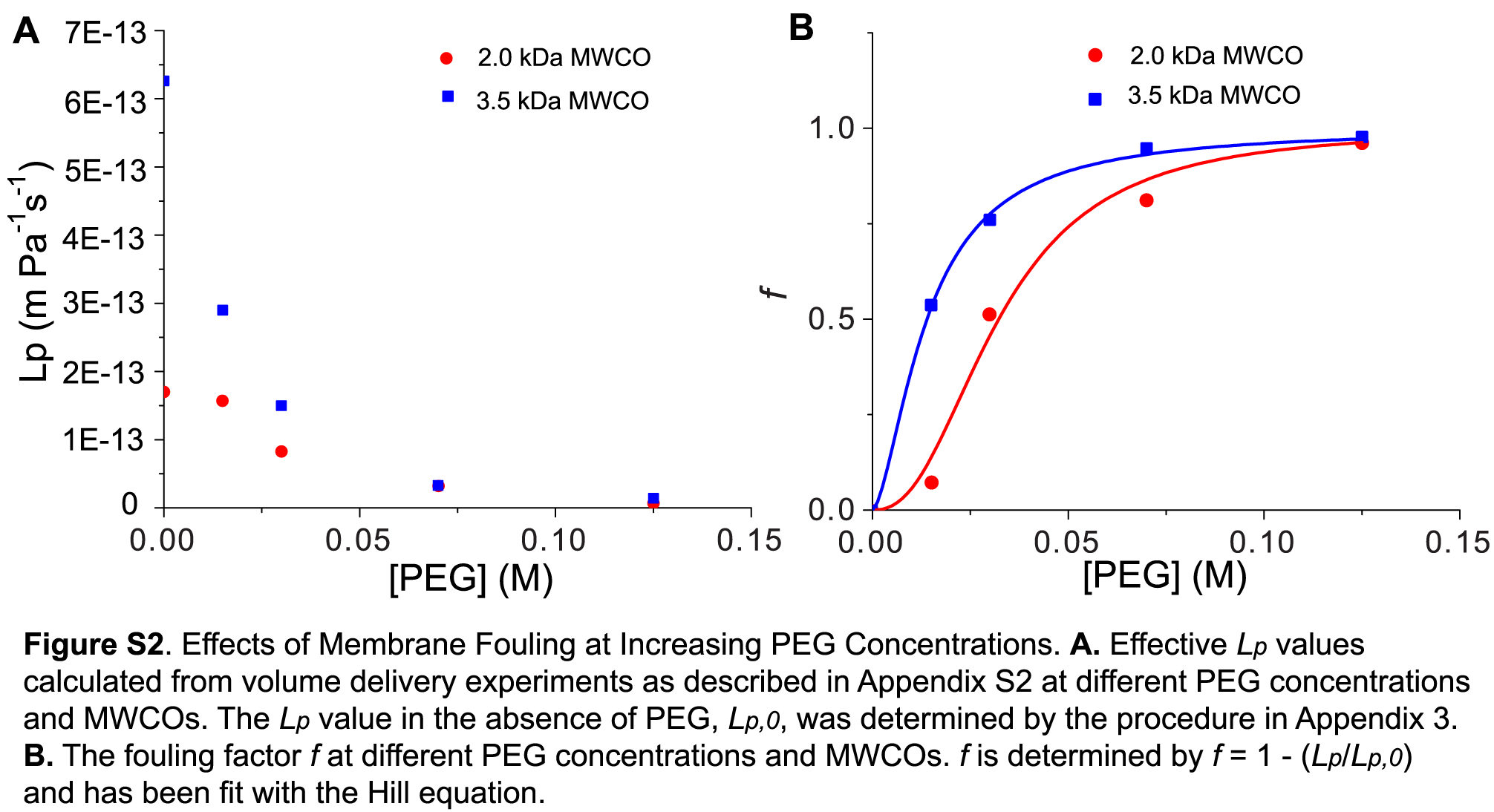

Supplement: Figure S2 — Effects of Membrane Fouling at Increasing PEG Concentrations. A. Effective Lp values calculated from volume delivery experiments as described in Appendix S2 at different PEG concentrations and MWCOs. The Lp value in the absence of PEG, Lp,0, was determined by the procedure in Appendix 3. B. The fouling factor f at different PEG concentrations and MWCOs. f is determined by and has been fit with the Hill equation. (TIF) [file pone.0091350.s002.tif]

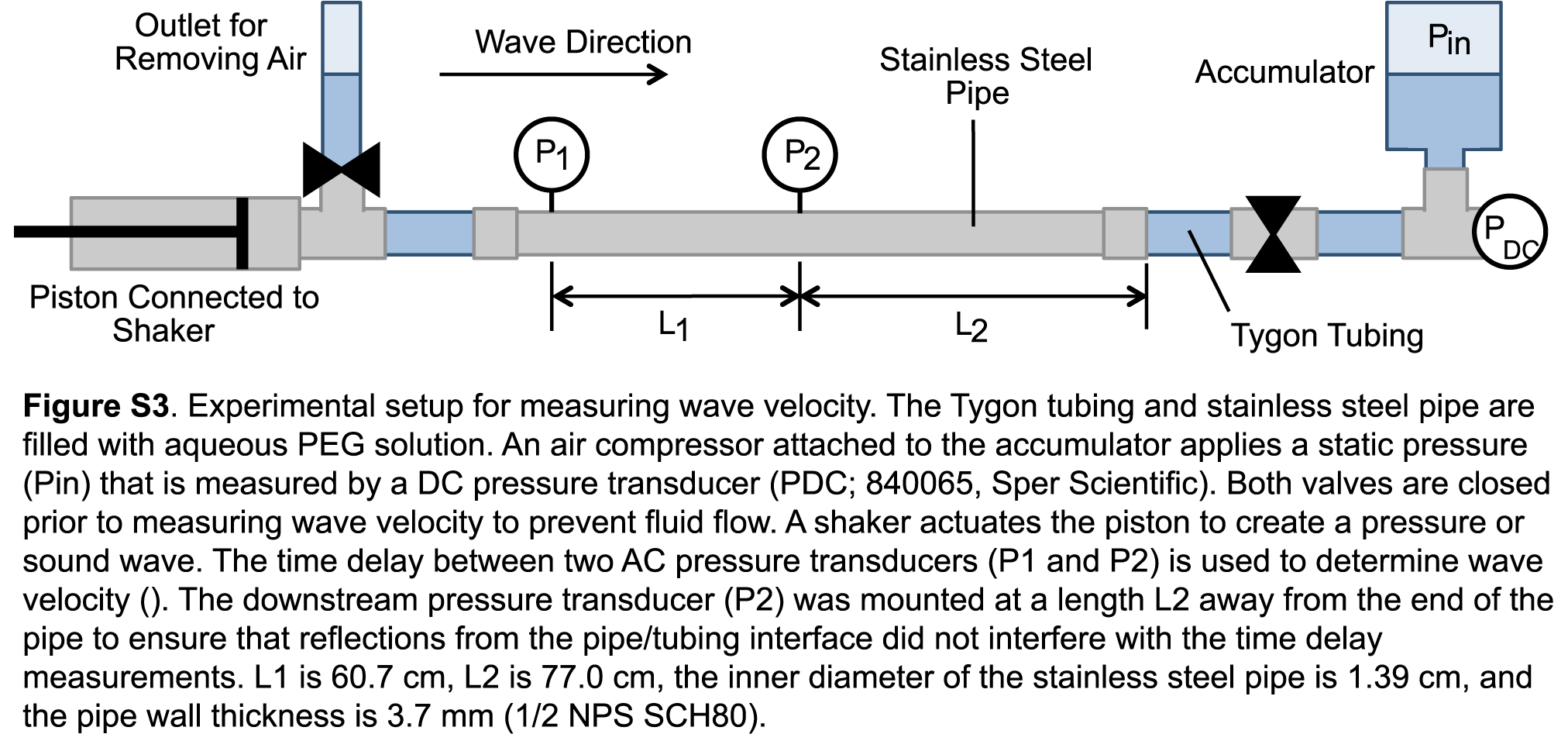

Supplement: Figure S3 — Experimental setup for measuring wave velocity. The Tygon tubing and stainless steel pipe are filled with aqueous PEG solution. An air compressor attached to the accumulator applies a static pressure (Pin) that is measured by a DC pressure transducer (PDC; 840065, Sper Scientific). Both valves are closed prior to measuring wave velocity to prevent fluid flow. A shaker actuates the piston to create a pressure or sound wave. The time delay between two AC pressure transducers (P1 and P2) is used to determine wave velocity (). The downstream pressure transducer (P2) was mounted at a length L2 away from the end of the pipe to ensure that reflections from the pipe/tubing interface did not interfere with the time delay measurements. L1 is 60.7 cm, L2 is 77.0 cm, the inner diameter of the stainless steel pipe is 1.39 cm, and the pipe wall thickness is 3.7 mm (1/2 NPS SCH80). (TIF) [file pone.0091350.s003.tif]

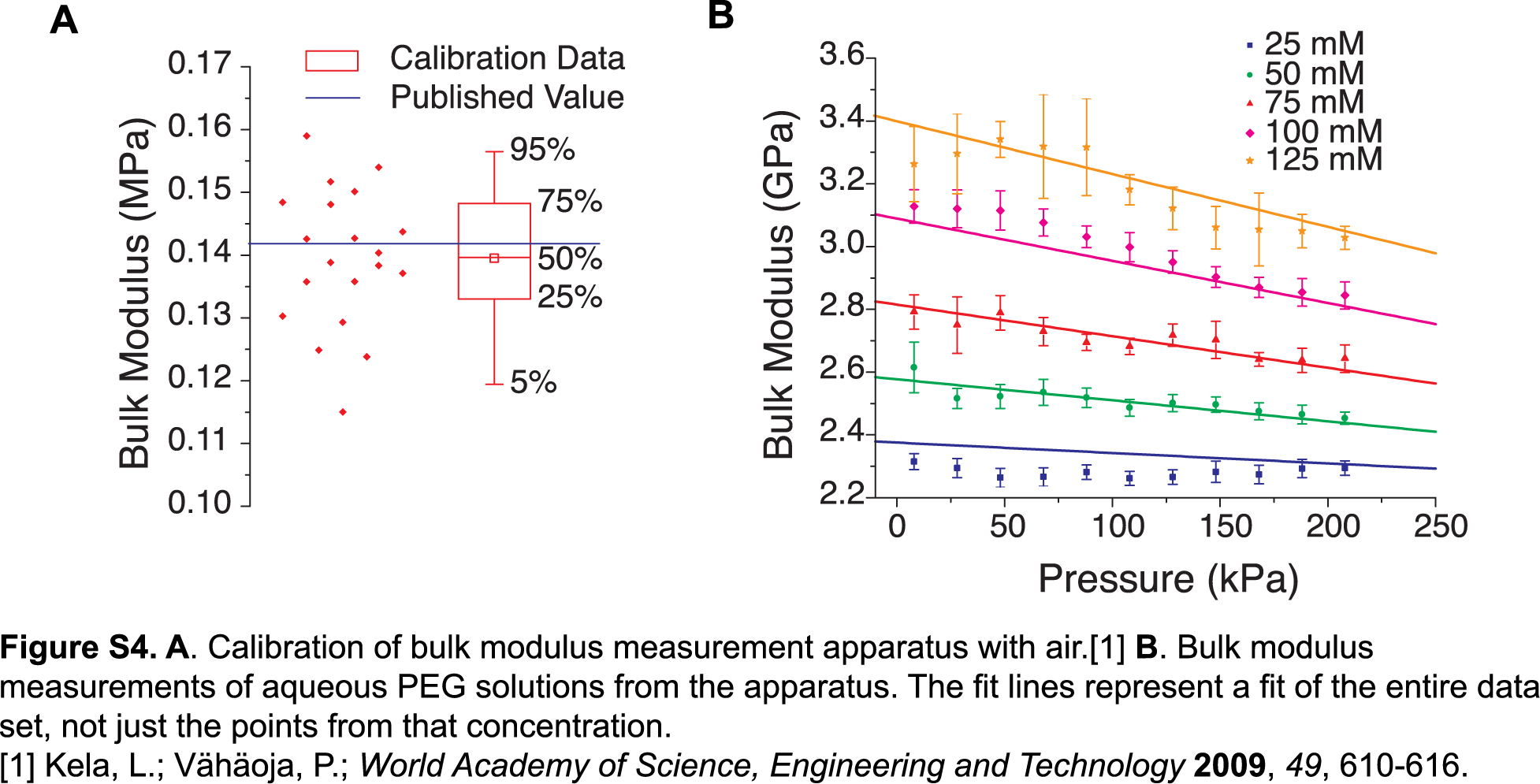

Supplement: Figure S4 — Bulk modulus calibration and measurements A. Calibration of bulk modulus measurement apparatus with air. B. Bulk modulus measurements of aqueous PEG solutions from the apparatus. The fit lines represent a fit of the entire data set, not just the points from that concentration. (TIF) [file pone.0091350.s004.tif]
