# Supplementary material for: Osmosis-Based Pressure Generation: Dynamics and Application
Source: PLoS One. 2014 Mar 10;9(3):e91350. doi: 10.1371/journal.pone.0091350 (PMC3948862; doi:10.1371/journal.pone.0091350)
Supplement: Appendix S1 — Osmotic Pressure Measurements. (DOCX) [file pone.0091350.s009.docx]

**Appendix S1. Osmotic Pressure Measurements**:

Measurements of the non-ideal osmotic character of aqueous PEG solutions were obtained from freezing point depression using an Advanced Instruments Model 3320 Micro-Osmometer. Curves were obtained from a cubic fit bound to the origin.

The osmometer is limited to taking measurements of species it is able to freeze, which restricts our measurements to concentrations of 80 mM and below. The function obtained from a cubic fit is:

$\Pi=\left(5.86 \text{Pa mol}^{3}\text{ m}^{-9}\right)*\left[PEG\right]^{3}+\left(196.94 \text{Pa mol}^{2}\text{ m}^{-6}\right)* \left[PEG\right]^{2}+\left(1634.14 \text{Pa mol m}^{-3}\right)*\left[PEG\right]$(N = 15, R^2^ > 0.99) (S1)

and is shown in Figure S1. $\Pi$ is measured in Pa, [*PEG*] is measured in mol m^-3^.
