# Supplementary material for: Osmosis-Based Pressure Generation: Dynamics and Application
Source: PLoS One. 2014 Mar 10;9(3):e91350. doi: 10.1371/journal.pone.0091350 (PMC3948862; doi:10.1371/journal.pone.0091350)
Supplement: Appendix S2 — Determining Lp from Volume Delivery. (DOCX) [file pone.0091350.s010.docx]

**Appendix S2. Determining *L_p_* from Volume Delivery**

A straightforward method for determining the hydraulic permeability (*L_p_*) of a dialysis cassette membrane is to perform a volume delivery experiment as described in the experimental section. Assuming minimal cassette expansion and/or pressure generation within the cassette, the first derivative of volume delivered with respect to time will equal the volumetric flow rate (*Q*) into the cassette, which is equal to *J_v_ * A*. These assumptions are more likely to be valid at the beginning of data collection as the influence of extenuating factors like imperfect mixing in the cassette upon influx of water, evaporation, and working fluid loss will grow over time.

From Equation 1 in the main text where *σ* = 1 and *ΔP* = 0, we obtain

$L_{p}= \frac{Q}{A *\Delta\Pi}$ (S2)

The membrane area, *A*, can be measured for each device. *Q* is equal to the initial slope of the volume delivered *vs.* time curve, which can easily be determined by fitting the initial portion of this data with a line after any artifacts resulting from cassette submersion have passed. *ΔΠ* can be determined from the initial concentration of the working fluid and the relation in section S1. The filtration coefficient, *L_p_*, can then be obtained from Equation S2.
