# Supplementary material for: Osmosis-Based Pressure Generation: Dynamics and Application
Source: PLoS One. 2014 Mar 10;9(3):e91350. doi: 10.1371/journal.pone.0091350 (PMC3948862; doi:10.1371/journal.pone.0091350)
Supplement: Appendix S3 — Discussion of Correction to Lp . (DOCX) [file pone.0091350.s011.docx]

**Appendix S3. Discussion of Correction to *L_p_***

The trend in effective *L_p_* observed from volume delivery experiments can be seen in Figure S2A. Processes involving semipermeable membranes often involve concentration polarization (CP) or membrane fouling; each of these conditions lowers the osmotic driving force and could explain the trend.[1]

First, we considered CP, of which there are four types. The conditions of our experiment led us to rule out some of these: we did not use an asymmetric membrane containing an active layer and a support layer, nor did we have an appreciable amount of solute in our “feed” solution.[2] With these considerations, the only type of CP that applies to our experiments is called dilutive external CP, in which the incoming water displaces the solute near the membrane inside the cassette. The correction that accounts for this involves multiplying the *ΔΠ* term in Equation 1 by a CP modulus:

$\frac{\pi_{\text{D},\text{m}}}{\pi_{\text{D},\text{b}}}=e^{\frac{-J_{v} d_{h}}{Sh D}}$ (S3)

For our experimental conditions, the CP modulus is approximately 1, meaning the effects of CP are negligible.[3] This agrees with an assertion made in Ullmann’s Encyclopedia of Industrial Chemistry that the laminar boundary layer inside which CP takes place in is too thin compared to the thickness of a dialysis membrane to have a strong effect.[1]

Membrane fouling effects involve blockage of flow via the accumulation of solute at the membrane surface and are complex, diverse, and difficult to model.[1] We decided to determine the *L_p_* for each MWCO in the absence of PEG (*L_p,0_*) by following the “Pumping Experiment for *L_p,0_* Determination” procedure in the Materials and Methods section of the main text. This yielded a flux value, $\frac{dz}{dt}$, which could be converted into an *L_p,0_* according to a modified version of Equation S2:

$L_{p,0}=\frac{\frac{dz}{dt} A_{tube}}{A_{mem}*\Delta P}$ (S4)

where *A_tube_* is the cross-sectional area of the thin tubing where the meniscus was monitored, *A_mem_* is the estimated total membrane area, and *ΔP* is the applied pressure.

As predicted, *L_p,0_* was higher than the effective *L_p_* values measured for both MWCO membranes in the presence of PEG (Figure S2A). As a result, we modeled the effective *L_p_* value as *L_p,0_*(1 – *f*), where *f* is a scaling factor that describes the decrease in effective *L_p_* with increasing PEG concentration. When plotted against PEG concentration, *f* was well-described by the Hill equation (R^2^ > 0.98), which describes cooperative binding of ligands to binding sites and takes the form $f= \frac{\left[PEG\right]^{n}}{\left[PEG\right]^{n} + k^{n}}$ (Figure S2B).[4] This adherence to a binding curve supports the hypothesis that the membrane is being fouled by PEG molecules, so we named *f* the “fouling factor.”

The best-fit values of the Hill equation fitting parameters for each MWCO are as follows:

2.0 kDa: *n* = 2.397, *k* = 0.322, R^2^ = 0.98, N = 5

3.5 kDa: *n* = 1.613, *k* = 0.0139, R^2^ = 0.999, N = 5

1. Strathmann H (2000) Membrane Separation Processes, 4. Concentration Polarization and Membrane Fouling. Ullmann’s Encyclopedia of Industrial Chemistry. Wiley-VCH Verlag GmbH & Co. KGaA. Available: http://onlinelibrary.wiley.com/doi/10.1002/14356007.o16_o05/abstract. Accessed 3 December 2013.

2. Cath TY, Childress AE, Elimelech M (2006) Forward osmosis: Principles, applications, and recent developments. J Membr Sci 281: 70–87. doi:10.1016/j.memsci.2006.05.048.

3. McCutcheon JR, Elimelech M (2006) Influence of concentrative and dilutive internal concentration polarization on flux behavior in forward osmosis. J Membr Sci 284: 237–247. doi:10.1016/j.memsci.2006.07.049.

4. Hill AV (1910) The possible effects of the aggregation of the molecules of haemoglobin on its dissociation curves. PROCEEDINGS OF THE PHYSIOLOGICAL SOCIETY: January 22, 1910. J Physiol 40: i–vii.
