# Supplementary material for: Osmosis-Based Pressure Generation: Dynamics and Application
Source: PLoS One. 2014 Mar 10;9(3):e91350. doi: 10.1371/journal.pone.0091350 (PMC3948862; doi:10.1371/journal.pone.0091350)
Supplement: Appendix S4 — Bulk Modulus Measurements. (DOCX) [file pone.0091350.s012.docx]

**Appendix S4. Bulk Modulus Measurements:**

The bulk modulus, *K* (Pa), of our working fluid is important because of its connection to density *ρ* (kg m^-3^), which varies with pressure according to:

$\rho=\frac{\rho_{atm}}{1+\frac{P_{in}}{K}}$ (S5)

where *ρ*_atm_ (kg m^-3^) is the fluid density at atmospheric pressure and *P_in_* is the gauge pressure of the device. The bulk modulus of aqueous PEG solutions of similar composition had previously only been derived as a function of concentration and not pressure.[1–7] Here, we characterized the bulk modulus of aqueous PEG-4000 solutions as a function of both concentration and pressure. To calculate bulk modulus, we measured the velocity of a pressure or sound wave traveling in solution and applied the following equation:

$K=\rho c^{2}$ (S6)

where *ρ* is the density of the solution and *c* is the wave velocity.[8] Substituting *ρ* with the expression shown in Equation S5 yields:

$K=\left(\frac{\rho_{atm}}{1+\frac{P_{in}}{K}}\right)c^{2}\to K=\rho_{atm}c^{2}+P_{in}\approx\rho_{atm}c^{2}$ for *P_in_* << *K* (S7)

where *ρ­_atm_* is the density of the solution at atmospheric pressure and *P_in_* is gauge pressure. We calculated *ρ­_atm_* for different PEG concentrations using an expression derived by Regupathi et. al.[9]

A schematic of the experimental setup used to measure wave velocity is shown in Figure S3. Briefly, a shaker (V408 shaker with PA100E power amplifier, Ling Dynamic Systems) generates a pressure wave that travels longitudinally along a rigid thick-walled stainless steel pipe under a no-flow condition. Two distantly spaced AC pressure transducers (101A06 sensor with Series 481A signal conditioner, PCB Piezotronics) are mounted flush with the pipe wall. An oscilloscope (DPO4032, Tektronix) records the output of each transducer at a sampling rate of 5 MHz. To determine wave velocity, the time delay between the transducers is calculated by using a custom-written MATLAB script. The script compares the initial portion of the two pressure waveforms, starting where each signal exceeds the RMS noise by 6 times the standard deviation and ending prior to the arrival of any reflections travelling in the upstream direction. To determine when such reflections will arrive at the downstream transducer (*P_2_*), we first estimated the wave velocity using the first 0.25 ms of each waveform. We assumed that the first reflections traveling upstream in the stainless steel pipe are generated at the interface between the pipe and Tygon tubing due to a change in cross-sectional area. Finally, the script compensates for attenuation of the pressure wave as it travels along the pipe by scaling the amplitude of the waveform of the downstream transducer such that the variance in time delay is minimized between the waveforms over the analysis region.

In order to test the method, we used it to measure the bulk modulus of air (Figure S3A). The value obtained, 0.14 MPa, is within 5% error of the published value. Bulk modulus values measured at six concentrations of PEG and a range of pressures from 0 to 208 kPa were fit to the following equation (Figure S4):

$K=2.89*10^{-5}*\left[PEG\right]^{2}+0.0059*\left[PEG\right]-1.35*10^{-5}*\left[PEG\right]*P_{in}+5.8*10^{-6}*P_{in}+2.21$ (S8)

where [*PEG*] is in mM, *P_in_* is pressure in Pa and *K* is bulk modulus in GPa. This correlation accounts for a second-order increase in K with [*PEG*] as well as a combinatorial effect of [*PEG*] and pressure.

1. Sasahara K, Uedaira H (1994) Volume and compressibility changes on mixing aqueous solutions of the amino acid and poly(ethylene glycol). Colloid Polym Sci 272: 385–392. doi:10.1007/BF00659449.

2. Rajulu AV, Sab PM (1995) Acoustical parameters of polyethylene glycol/water mixtures. Bull Mater Sci 18: 247–253.

3. Jannelli MP, Magazu S, Maisano G, Majolino D, Migliardo P (1994) Non-ideal compressibility in poly (ethylene oxide)—water solutions induced by H-bond interactions. J Mol Struct 322: 337–343.

4. Palani R, Balakrishnan S, Roomy AMS (2010) Acoustical and thermodynamical properties of PEG in non-electrolytes at 303, 313 and 323 K. Arch Phys Res 1: 111–118.

5. Żwirbla W, Sikorska A, Linde BBJ (2005) Ultrasonic investigations of water mixtures with polyethylene glycols 200, 400 and ethylene glycol. J Mol Struct 743: 49–52. doi:10.1016/j.molstruc.2005.02.019.

6. Rajulu AV, Sab PM (1996) Ultrasonic studies of water/poly (ethylene glycol) mixtures. Eur Polym J 32: 267–268.

7. Sasahara K, Sakurai M, Nitta K (1998) Volume and compressibility changes for short poly (ethylene glycol)–water system at various temperatures. Colloid Polym Sci 276: 643–647.

8. Kela L, Vähäoja P (2009) Measuring pressure wave velocity in a hydraulic system. Proc World Acad SET 37: 610–616.

9. Regupathi I, Murugesan S, Amaresh SP, Govindarajan R, Thanabalan M (2009) Densities and Viscosities of Poly(ethylene glycol) 4000 + Diammonium Hydrogen Phosphate + Water Systems. J Chem Eng Data 54: 1100–1106. doi:10.1021/je800769c.
