# Supplementary material for: Osmosis-Based Pressure Generation: Dynamics and Application
Source: PLoS One. 2014 Mar 10;9(3):e91350. doi: 10.1371/journal.pone.0091350 (PMC3948862; doi:10.1371/journal.pone.0091350)
Supplement: Appendix S5 — Determination of Vin ( Pin ). (DOCX) [file pone.0091350.s013.docx]

**Appendix S5. Determination of *V_in_*(*P_in_*)**

*General Procedure*: The dialysis cassette and pressure transducer were prepared as described in the Experimental Section, although water was used as the working fluid and the cassettes were initially filled to their advertised capacity (3 or 30 mL). A syringe filled with Milli-Q water was fitted with rigid tubing and a needle was connected to the submerged dialysis cassette. A syringe pump was used to compress the syringe at a rate of 1 mL/minute; simultaneously, pressure was recorded. The procedure was repeated for each cassette and restraint condition. Recording continued until cassette failure. The line representing *V_in_*(t) was plotted against *P_in_*(t) and fitted with a fifth-order polynomial representing *V_in_*(*P_in_*).

*Curves*:

0.5-3 mL, 2 kDa MWCO cassettes, restrained:

$$\begin{matrix} V_{in}=\left(4.4214*10^{-6} \text{m}^{3}\right)+\left(8.04609*10^{-11} \text{m}^{3} \text{Pa}^{-1}\right)P_{in}+\left(-3.07174*10^{-15} \text{m}^{3} \text{Pa}^{-2}\right)P_{in}^{2} \\ +\left(6.68335*10^{-20} \text{m}^{3} \text{Pa}^{-3}\right)P_{in}^{3}+\left(-7.40082*10^{-25} \text{m}^{3} \text{Pa}^{-4}\right)P_{in}^{4}+\left(3.40544*10^{-30} \text{m}^{3} \text{Pa}^{-5}\right)P_{in}^{5} \end{matrix}$$

0.5 – 3 mL, 3.5 kDa MWCO cassettes, restrained:

$$\begin{matrix} V_{in}=\left(5.7711*10^{-6} \text{m}^{3}\right)+\left(3.69131*10^{-11} \text{m}^{3} \text{Pa}^{-1}\right)P_{in}+\left(-1.16582*10^{-15} \text{m}^{3} \text{Pa}^{-2}\right)P_{in}^{2} \\ +\left(2.1355*10^{-20} \text{m}^{3} \text{Pa}^{-3}\right)P_{in}^{3}+\left(-1.6605*10^{-25} \text{m}^{3} \text{Pa}^{-4}\right)P_{\text{i}n}^{4}+\left(4.73849*10^{-31} \text{m}^{3} \text{Pa}^{-5}\right)P_{in}^{5} \end{matrix}$$

0.5-3 mL, 2 kDa MWCO cassettes, unrestrained:

$$\begin{matrix} V_{in}=\left(6.03765*10^{-6} \text{m}^{3}\right)+\left(1.69831*10^{-11} \text{m}^{3} \text{Pa}^{-1}\right)P_{in}+\left(-6.64465*10^{-15} \text{m}^{3} \text{Pa}^{-2}\right)P_{in}^{2} \\ +\left(1.93811*10^{-19} \text{m}^{3} \text{Pa}^{-3}\right)P_{in}^{3}+\left(-2.65978*10^{-24} \text{m}^{3} \text{Pa}^{-4}\right)P_{in}^{4}+\left(1.38578*10^{-29} \text{m}^{3} \text{Pa}^{-5}\right)P_{in}^{5} \end{matrix}$$

0.5-3 mL, 3.5 kDa MWCO cassettes, restrained:

$$\begin{matrix} V_{in}=\left(4.49345*10^{-6} \text{m}^{3}\right)+\left(7.50511*10^{-10} \text{m}^{3} \text{Pa}^{-1}\right)P_{in}+\left(-7.76213*10^{-14} \text{m}^{3} \text{Pa}^{-2}\right)P_{in}^{2} \\ +\left(5.07533*10^{-18} \text{m}^{3} \text{Pa}^{-3}\right)P_{in}^{3}+\left(-1.55112*10^{-22} \text{m}^{3} \text{Pa}^{-4}\right)P_{in}^{4}+\left(1.85547*10^{-27} \text{m}^{3} \text{Pa}^{-5}\right)P_{in}^{5} \end{matrix}$$

For all curves, *P_in_* is measured in Pa, *V_in_* is measured in m^3^ and R^2^ > 0.999.
